# Long-Circulating and Brain-Targeted Liposomes Loaded with Isoliquiritigenin: Formation, Characterization, Pharmacokinetics, and Distribution

**DOI:** 10.3390/pharmaceutics16080975

**Published:** 2024-07-24

**Authors:** Weitong Song, Lu Bai, Pingxiang Xu, Yuming Zhao, Xuelin Zhou, Jie Xiong, Xiaorong Li, Ming Xue

**Affiliations:** 1 Department of Pharmacology, School of Basic Medical Sciences, Capital Medical University, Beijing 100069, China; 2 Beijing Laboratory for Biomedical Detection Technology and Instrument, Capital Medical University, Beijing 100069, China

**Keywords:** isoliquiritigenin, nanoliposomes, angiopep-2, brain distribution

## Abstract

Isoliquiritigenin (ISL) has excellent neuroprotective effects. However, its limitations, including poor solubility, low bioavailability, and low accumulation in the brain, restrict its clinical promotion. In this study, a novel type of ISL-loaded liposome (ISL-LP) modified with the brain-targeting polypeptide angiopep-2 was prepared to improve these properties. The zeta potential, morphology, particle size, encapsulation efficiency, drug loading, and in vitro release of ISL-LP were evaluated. The pharmacokinetics and tissue distribution of ISL and ISL-LP were also investigated. The results demonstrated that ISL-LP had an average particle size of 89.36 ± 5.04 nm, a polymer dispersity index of 0.17 ± 0.03, a zeta potential of −20.27 ± 2.18 mV, and an encapsulation efficiency of 75.04 ± 3.28%. The in vitro release experiments indicate that ISL-LP is a desirable sustained-release system. After intravenous administration, LPC-LP prolonged the circulation time of ISL in vivo and enhanced its relative brain uptake. In conclusion, ISL-LP could serve as a promising brain-targeting system for the treatment and prevention of central nervous system (CNS) disorders.

## 1. Introduction

Licorice is considered a traditional Chinese medicine. In China, the earliest written reference to licorice is found in Shen Nong Ben Cao Jing, the first Chinese dispensary. Due to its extensive pharmacological activity, licorice has been used for centuries as a reconciler in Chinese herbal compounds [1]. As modern pharmacology has developed, licorice has been found to have powerful effects, mainly due to its various natural active ingredients, among which isoliquiritigenin (ISL) is the main component [2]. ISL is a flavonoid-active compound isolated from the root of licorice. It is usually used as a main marker for the chemical evaluation of licorice and its products. With the increasing research on the mechanism of ISL in recent years, it has been discovered that this compound exhibits a wide range of effects, including anti-oxidant, anti-cancer, anti-viral, anti-inflammatory, and immune regulation [3]. In addition to the pharmacological activities mentioned above, a multitude of studies have demonstrated that ISL has significant neuropharmacological activities. For instance, X. Ma et al. found that ISL had a protective effect on learning and memory impairments induced by a high-fat diet through the inhibition of the TNF-α/JNK/IRS pathway [4]. Several studies revealed that ISL has valuable pharmacological activities against age-related neurodegenerative diseases and disorders (NDDs), such as Alzheimer’s disease (AD) and Parkinson’s disease (PD) [5]. Notably, ISL has been found to exhibit a significant inhibitory effect on glioma cells such as SHG44 [6], U251 [7], U87, and C6 cells [8]. The anti-cancer mechanism of ISL against glioma is multifaceted, mainly including induction of autophagy, promotion of apoptosis, influence on the cell cycle, induction of cell differentiation, inhibition of cell proliferation, inhibition of metastasis and invasion of tumor cells, and resistance to multidrug resistance in tumor cells. Accordingly, isoliquiritigenin is a multi-mechanism, multi-channel, multi-target anti-glioma drug. Furthermore, numerous studies in vitro and in vivo have demonstrated that ISL exerts a neuroprotective effect [9]. It is therapeutically efficacious for cerebral ischemic injury and cerebral ischemia-reperfusion injury caused by various reasons [10]. Based on these findings, there is no doubt that ISL is a promising drug for the treatment and prevention of various neurological disorders in the human brain. However, the clinical promotion of ISL is limited by the drawbacks of isoliquiritigenin’s physicochemical properties, such as its low aqueous solubility and low release efficiency in vitro. In addition, pharmacokinetic investigations have also demonstrated that ISL has serious limitations, including low oral absorption, a short half-life in vivo, poor bioavailability, and low brain distribution [11]. As a result, the cerebral protective effects of isoliquiritigenin are significantly limited by these drawbacks.

The ability of a drug to penetrate the blood–brain barrier (BBB) is a critical determinant in evaluating its effectiveness as a neuroprotective agent for the treatment of cerebral disorders. The blood–brain barrier is comprised of tightly connected structures, including endothelial cells, basement membrane, pericytes, and astrocytes, which are situated between brain tissue and brain capillaries. The maintenance of the normal physiological state in the central nervous system is dependent on this critical structure [12]. Simultaneously, the presence of the BBB limits the passage of almost all medicines from the bloodstream to the cerebral tissue, thereby greatly affecting the effectiveness of pharmaceuticals in treating neurological disorders. In order to enhance the targeted delivery of drugs to the brain, researchers have developed a number of strategies, including chemical modification of the drug or its prodrug, temporary disruption of the tight junctions of the BBB, neurosurgical interventions, nanotechnology-mediated drug delivery, etc. [13]. Among the various strategies, the utilization of nanoparticles to facilitate drug delivery across the BBB emerges as a comparatively effective, safe, and non-invasive approach.

As the field of nanomedicine has developed over the past few decades, a range of delivery systems have been utilized to overcome the aforementioned physicochemical constraints of drugs, improve the targeting of specific organs, and control the release kinetics of drugs within the body. Various delivery systems, such as mesoporous silicas, liposomes, polymer micelles, dendrimers, and others, have been investigated [14,15,16,17,18]. Among them, liposomes have gained significant attention as a promising drug delivery system owing to their convenient preparation, capacity to improve water solubility and bioavailability, minimal toxicity, high lipophilicity, and favorable biocompatibility [19]. The main constituents of liposomes were phosphatidylcholine (PC) and cholesterol (Chol), which are the natural components of cell membranes and lipoproteins [20]. After entering the circulatory system, liposomes are subjected to various factors, such as geometry, particle size, and charge characteristics. As a result, they may be readily recognized as foreign bodies by macrophages and subsequently phagocytosed. This process can lead to the accumulation of liposomes in diverse organs and tissues, including the brain, liver, and spleen. Accordingly, liposomes possess an inherent ability to overcome obstacles posed by the BBB while simultaneously maintaining structural integrity. This allows them to enhance drug concentration in the brain, thereby exhibiting passive targeting. Various modifications can be employed on the surfaces of liposomes for different purposes. Polyethylene glycol (PEG)-modified liposomes offer increased potential for passive targeting. PEG is a hydrophilic polymer that enhances the structural stability of liposomes. It plays a crucial role in preventing clearance by the mononuclear phagocyte system (MPS) and prolonging drug circulation in vivo [21]. The properties of PEG facilitate the enhanced accumulation of drug concentrations in specific tissues. In our laboratory, we prepared and investigated PEGylated liposomes loaded with lapachol, a therapeutic agent used for the treatment of glioma, through in vivo and in vitro experiments to assess the properties and efficacy of these liposomes [22]. The investigation revealed that PEGylated liposomes can effectively alter the physicochemical characteristics of lapachol, leading to an improved pharmacokinetic profile within the body. Furthermore, these liposomes facilitate the uptake of lapachol in the brain, thereby enhancing its anti-glioma effects. These findings indicate that PEGylated liposomes exhibit significant promise as nanoparticles for the treatment of gliomas [22]. In addition, it is possible to modify targeting ligands on the surface of liposomes. The ligand induces the endocytosis of nanoparticles by binding to specific receptors on the parietal membrane of brain endothelial cells. This facilitates the targeted accumulation of drugs in the central nervous system, while simultaneously reducing the adverse impact of the medication on non-target organs. Angiopep-2 (ANG), a 19-amino acid active target peptide, exhibits a high affinity for the low-density lipoprotein receptor-related protein-1 (LRP-1) on the surfaces of the vascular endothelial cells of the BBB. This interaction significantly facilitates the penetration of nanoparticles across the BBB [23]. LRP-1 can mediate the endocytosis of several ligands to cross the BBB, such as lactoferrin (LF), transferrin (TF), ANG, etc. [24]. Nanoformulations modified with TF or LF compete with natural ligands for the same receptors, which are largely saturated with endogenous TF or LF, resulting in low targeting efficiency. In contrast, ANG is non-endogenous and exhibits greater penetration efficiency across the BBB compared to LF and TF. Consequently, it is favored by an increasing number of researchers [25]. In the previous stage, we have successfully synthesized DSPE-PEG2000-Angiopep-2 by attaching Angiopep-2 to the terminal end of DSPE-PEG2000-Mal in our laboratory [26].

**Figure 1:**
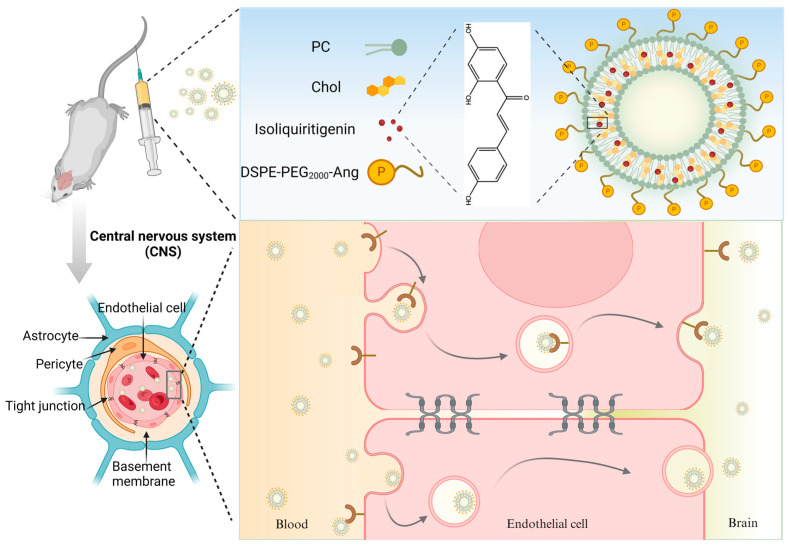
Preparation of ISL-LP and the process of delivering ISL-LP to brain tissue across the BBB.

ISL exhibits promising potential for the treatment and prevention of brain diseases. However, the physicochemical deficiency limits its clinical application. The purpose of this investigation is to obtain a nanoparticle loaded with ISL that exhibits enhanced stability, improved bioavailability, and the ability to specifically target the brain. In this study, ISL-loaded PEGylated liposomes modified with ANG were prepared using the membrane dispersion method for the first time. The formulations were subsequently evaluated for various physical properties such as appearance, particle size, polydispersity index, zeta potential, etc. Additionally, in vitro release studies were also carried out in different media. Finally, we studied the pharmacokinetics and tissue distribution of ISL nanoparticles in SD rats and ICR mice, with ISL suspension as the control. In conclusion, the experimental results indicate that ISL-LP is a novel and effective candidate for the treatment and prevention of brain diseases, and it provides an efficient drug delivery strategy for brain targeting. Figure 1 illustrates the preparation of ISL-LP and the process of delivering ISL-LP to brain tissue across the BBB, created using BioRender.com (accessed on 27 June 2024).

## 2. Materials and Methods

### 2.1. Materials and Animals

Isoliquiritigenin (purity > 98%) was obtained from Chengdu Must Bio-Technology Co., Ltd. (Chengdu, China). Tris (2-carboxyethyl) phosphine (TCEP, purity > 98%) was provided by Beijing Solarbio Science and Technology Co., Ltd. (Beijing, China). Angiopep-2 (purity > 98%) was purchased from Shanghai Top Biological Technology Co., Ltd. (Shanghai, China). DSPE-PEG2000-Mal (purity > 98%) [1,2-distearoyl-sn-glycero-3-phosphoethanolamine-N-maleimide (polyethylene glycol)-2000] was bought from Shanghai Ponsure Biotechnology Co., Ltd. (Shanghai, China). Chol and PC (purity > 99%) were purchased from ABCONE (Shanghai, China). HPLC-grade acetonitrile (purity > 99.99%) was obtained from Fisher Scientific (Fair Lawn, NJ, USA). All other chemical reagents employed in this study were of analytical grade.

SD rats (250 ± 30 g, male) and ICR mice (25 ± 2 g, male) raised in the Animal Center of Capital University of Medical Sciences (Beijing, China) were housed in a controlled environment at a temperature of 25 ± 1 °C for 5 days. Before the experiment, the animals were fasted for 12 h without access to water. All procedures of the animal experiment are in accordance with the Regulations of the Experimental Animal Administration.

### 2.2. Preparation of Solutions

To prepare the ISL methanol solution, a precise quantity of ISL was weighed and then dissolved in methanol to achieve a concentration of 2 mg/mL. For the Chol and PC solution, phosphatidylcholine and cholesterol were dissolved in dichloromethane to form a 1 mg/mL solution. A precise amount of DSPE-PEG2000-Angiopep-2 was added to a test tube with chloroform and then sonicated at room temperature for 30 min to prepare a 1 mg/mL DSPE-PEG2000-Angiopep-2 solution. All solutions were stored in a refrigerator at a temperature of −20 °C.

### 2.3. Preparation of ISL-LP

The ISL-LP was prepared using the thin-film rotary evaporation method, as previously described with certain modifications [22]. Briefly, the appropriate amounts of ISL methanol solution, DSPE-PEG2000-Angiopep-2 chloroform solution, and Chol and PC dichloromethane solutions were mixed in a distillation flask, followed by agitation and sonication for ten minutes. Subsequently, the organic solvent of the mixed solution was removed with vacuum rotary evaporation at a temperature of 45 °C and a rotating speed of 130 rpm min^−1^ for 20 min to ensure complete evaporation. After obtaining the thin film, it was rehydrated with 5 mL of distilled water under the same experimental conditions for 30 min. Afterward, the solution was sonicated for 10 min and filtered through an organic filter membrane of 0.22 μm to remove the free ISL and obtain the yellow ISL-LP solution.

### 2.4. Optimization of the ISL-LP Formulation

In order to optimize the formulation of ISL-LP, a three-factor, three-level orthogonal test was conducted to investigate the effect of the dosage and ratio of ISL, PC, and Chol on the encapsulation efficiency (EE) of liposomes. EE is a critical indicator for the quality control of liposomes, as it reflects the extent of drug encapsulation within the liposome. The three factors included the amount of PC (factor A), the PC: ISL quality ratio (factor B), and the PC & Chol: ISL quality ratio (factor C). Three levels were examined for each factor, as shown in Table 1. Therefore, nine experiments were conducted to determine the final formulation, with EE serving as an evaluation indicator.

### 2.5. Characterization of ISL-LP

A total of 100 µL of freshly prepared ISL-LP nanoparticles was diluted 50 times with distilled water and sonicated at room temperature for 20 min. Then, 1 mL of the solution was added to the folded capillary zeta cell (DTS1070) and quartz cuvette in the Zetasizer Nano S90 (Malvern Instruments, Malvern, UK) to detect the zeta potential (ZP) and particle size of LPC-LP. The measurements were conducted three times in parallel.

For the morphology of ISL-LP, a drop of diluted ISL-LP solution was placed on a copper grid coated with carbon and then negatively stained with phosphotungstic acid. After the solution was dried at room temperature, the micromorphology of ISL-LP was observed and photographed by a transmission electron microscope (JEM-2010, Hitachi, Tokyo, Japan).

A total of 100 μL of ISL-LP solution was added to 900 μL of methanol and then subjected to vortex shaking for 10 min to disrupt the liposome structure. Following centrifugation at a speed of 13,000 rpm and a temperature of 4 °C for 10 min, 100 μL of the supernatant was diluted with methanol and then filtered through 0.22 μm membranes. Subsequently, 15 μL of the solution was injected into a high-performance liquid chromatography system (HPLC, Agilent 1100, Santa Clara, CA, USA) to detect the concentration of ISL according to the method previously reported [26]. Before these steps, we quantified the concentration of free isoliquiritigenin in the saturated aqueous phase by HPLC. It was found that the concentration of isoliquiritigenin was below the detection limit of our HPLC system, confirming that the residual amount of unentrapped drug in the aqueous phase was negligible and could not affect the overall analysis. The drug loading (DL) and EE of ISL-LP were calculated according to Equations (1) and (2):

EE (%) = (W_loaded_/W_added)_ × 100% (1)

DL (%) = (W_loaded_/W_total_) × 100% (2)

where W_loaded_ represents the amount of ISL loaded, W_added_ denotes the weight of ISL added, and W_total_ signifies the weight of liposomes. 

### 2.6. In Vitro Release Studies

The dialysis bag diffusion technique was employed to study the in vitro release of ISL from ISL-LP. The appropriate amount of sodium dodecyl sulfate (SDS) was weighed and subsequently dissolved in phosphate-buffered saline (PBS) to prepare the PBS solution containing 1% SDS. A free ISL solution was prepared by dissolving an appropriate amount of ISL in 1% SDS-PBS. Afterward, an appropriate amount of 1% SDS-PBS was taken, and its pH was adjusted to 7.4 as a release medium. A total of 2 mL of ISL-LP and free ISL solution (300 μg/mL) were transferred into dialysis bags (MWCO: 1000). The dialysis bag was fastened at both ends with thin wire and then placed in a beaker containing 50 mL of release media. Throughout the experiment, the beaker was firmly attached to thermostatically controlled vibrators to maintain a constant temperature of 37 °C and shaken at a speed of 200 rpm. A total of 200 μL of the solution was removed at 0.5, 1, 2, 4, 6, 8, 12, 24, and 48 h from the beakers for subsequent HPLC analysis of ISL. Simultaneously, an equivalent volume of fresh release solution at the same temperature was replenished at each time point to maintain sink conditions. The experiments were conducted in triplicate for each experimental group.

### 2.7. Pharmacokinetics of ISL-LP and ISL

Twelve male SD rats with jugular vein cannulation were randomly divided into two groups. The injection solution of ISL-LP was prepared as described above. The ISL suspension was obtained by dissolving ISL in normal saline containing 20% PEG400 and 30% propanediol. After weighing, the rats were administered ISL-LP and ISL suspension by tail vein injection at a dosage of 2 mg/kg. A total of 300 μL of blood was withdrawn from the jugular cannula at 2, 5, 10, 30, 60, 120, 240, and 480 min after intravenous administration and then transferred into pre-heparinized EP tubes. Meanwhile, an equal volume of saline was injected into the rat through the jugular vein cannula immediately following each blood sample collection. The blood samples collected were subsequently centrifuged at a speed of 13,000 rpm for 10 min. The supernatant was transferred into Eppendorf tubes and subsequently stored at −80 °C for further processing.

The plasma was processed as described in previous reports [26]. Briefly, 100 μL of the internal standard (genistein, 1 μg/mL) and 300 μL of acetonitrile were mixed with the plasma. The mixture was vortexed for two minutes to completely precipitate the protein. After centrifuging for 10 min at 10,000 rpm, 400 μL of the supernatant was transferred to the tubes and then dried by a vacuum concentrator. The dried sediments were redissolved in 200 μL of HPLC-grade methanol. After vortexing for 5 min and centrifuging at 13,000 rpm for 10 min, the supernatant was filtered through membranes (0.22 μm) and analyzed by UPLC-MS/MS to detect ISL according to the method previously reported [26].

The pharmacokinetic statistical parameters of ISL from ISL-LP and ISL suspension in SD rats were calculated by the DAS 2.0 pharmacokinetics software.

### 2.8. Tissue Distribution Study

A total of thirty-two ICR mice were used for the investigation of tissue distribution. After recording the body weight, the mice were randomly divided into 2 groups, with 16 mice in each group. The ISL-LP and ISL suspension were injected intravenously to mice at a dose of 2.8 mg/kg. At time intervals of 10, 30, 60, and 120 min after administration, four mice from each group were sacrificed by decapitation. The tissues (brain, lung, heart, liver, and kidney) were collected, rinsed with cold normal saline, wiped with filter paper, and weighed immediately. Subsequently, the tissue was homogenized in cold normal saline and finally prepared as a homogenate solution with a concentration of 0.5 g/mL. After centrifuging at 13,000 rpm for 10 min, the supernatant was transferred to a tube and stored at −80 °C before analysis. The tissue samples were subjected to the same treatment and analysis as the plasma samples described above.

In order to evaluate the targeting efficiency of ISL-LP in vivo compared with ISL suspension, several targeting indicators were calculated in this study, such as targeting efficiency (TE), targeting index (TI), and relative targeting efficiency (RTE) [27]. The area under the concentration–time curve (AUC) of ISL in different tissues was determined by the linear trapezoidal method [28]. TE, TI, and RTE are calculated from Equations (3)–(5):

(3) $$TE=\frac{{\left({AUC}_{0\to \infty }\right)}_{i}}{\sum_{i=1}^{n}{\left({AUC}_{0\to \infty }\right)}_{i}}\times 100\%$$

(4) $$TI=\frac{{\left({AUC}_{0\to \infty }\right)}_{ISL-LP}}{{\left({AUC}_{0\to \infty }\right)}_{ISL}}$$

(5) $$RTE=\frac{{TE}_{ISL-LP}-{TE}_{ISL}}{{TE}_{ISL}}\times 100\%$$

where the numerator and denominator of TE represent the AUC of a certain tissue and the total exposure of ISL in all tissues, respectively. (AUC_0→∞_)_ISL-LP_ and (AUC_0→∞_)_ISL_ refer to the AUC of ISL in a specific tissue after administration of ISL-LP and ISL suspension, while TE_ISL-LP_ and TE_ISL_ denote the targeting efficiency of ISL-LP and ISL in the same tissues.

### 2.9. Statistical Analysis

All the data was presented as means ± standard deviation (SD). At least three independent measurements were conducted under identical conditions to confirm all the experiments. The statistical analysis was performed with SPSS 19.0 statistical software. The groups were analyzed by a one-way ANOVA, and a *p*-value less than 0.05 was considered statistically significant.

## 3. Results and Discussion

### 3.1. Preparation and Optimization of the LPC-LP Formulation

In the present study, DSPE-PEG2000-Angiopep-2-liposomes loaded with ISL were synthesized for the first time using the thin-film rotary evaporation method, according to Section 2.3. Given the main constituents of liposomes, the optimization of the formulation primarily focused on the dosage and ratio of PC, Chol, and ISL. The L9 (3^4^) orthogonal test was designed with EE% as the evaluation index to optimize the formulation of ISL-LP. The results of nine formulations are presented in Table 2. According to the order of extreme values (A > C > B), the amount of PC has the greatest impact on the EE of ISL-LP, followed by the ratio of (PC + Chol) to ISL, and then the ratio of PC to ISL. The results were consistent with the variance analysis. The amount of PC and the ratio of (PC + Chol) to ISL have a statistically significant effect on the EE of ISL-LP (*p* < 0.05), whereas the ratio of PC to ISL has no statistically significant effect. The influence of different levels for each factor on the EE of ISL-LP was described as follows: A3 > A2 > A1; B2 > B3 > B1; C2 > C3 > C1, as shown in Table 2. Based on the results, it can be determined that the optimal synthesis formulation for ISL-LP is A3B2C2, i.e., the amount of PC was 9 mg, the ratio of PC to LPC was 6:1, and the ratio of (PC + Chol) to ISL was 20:1.

### 3.2. Characterization of ISL-LP

**Figure 2:**
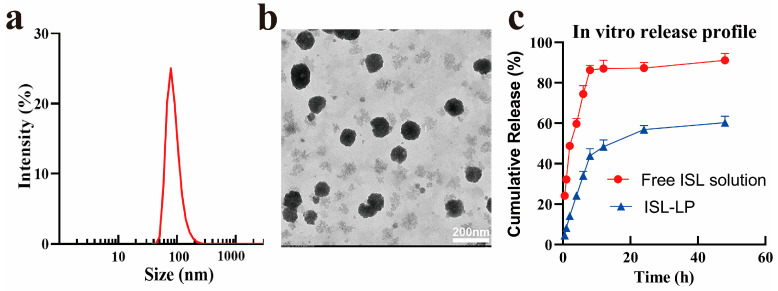
(**a**) The particle size distribution of ISL-LP. (**b**) The TEM image of ISL-LP. (**c**) In vitro release profiles of free ISL solution and ISL-LP in PBS at pH = 7.4.

Three batches of ISL-LP were prepared according to the optimal formulation, as previously described. The particle size, polydispersity index (PDI), and ZP of LPC-LP were measured by the Zetasizer Nano S90 instrument, while the size distributions and morphology were observed by the transmission electron microscope (TEM). The results are presented in Figure 2. The encapsulation efficiency and drug loading of ISL-LP were 75.04 ± 3.28% and 3.31 ± 0.30%, respectively. The average particle size of the optimized formulation was 89.36 ± 5.04 nm, with a narrow PDI of 0.17 ± 0.03 and a zeta potential of −20 ± 2.18 mV. The TEM images revealed that the ISL-LP particles are spherical and uniformly distributed in an aqueous solution. The stability of nanoparticles in a solution and their behavior in vivo are determined by their physical properties. Previous studies have demonstrated that liposomes with a particle size ranging from 50 to 100 nm possess the ability to avoid phagocytosis by the reticuloendothelial system [29]. Additionally, these liposomes decrease plasma clearance and prolong the duration of drugs in the body. Furthermore, it has been observed that nanomedicines with a particle size smaller than 100 nm are easier to penetrate the vascular endothelial cells of the BBB through endocytosis [30]. The ISL-LP exhibits an average particle size of 89.36 ± 5.04 nm, suggesting that the LPC-LP will likely achieve similar pharmacokinetic advantages, including prolonged circulation time and enhanced drug delivery to the brain. The PDI is employed to evaluate the size distribution of nanoparticles and is considered an indicator of the uniformity of the distribution. In general, a PDI value around 0.1 indicates high homogeneity across the particle population, whereas a high PDI value may suggest a wide size distribution or even multiple populations [31]. The narrow PDI of 0.17 ± 0.03 for LPC-LP indicates a relatively uniform size distribution. This uniformity can minimize the variability in the pharmacokinetics and distribution of LPC-LP in vivo. The shape of nanoparticles also affects the cellular uptake of drugs. Spherical nanoparticles are more easily absorbed by cells than non-spherical nanoparticles [32,33]. Additionally, they have been shown to reduce the irritation of nanoparticles to blood vessels during intravenous administration [34]. Therefore, the spherical shape of ISL-LP provides a distinct advantage in terms of enhancing the therapeutic efficacy and safety profile of the drug delivery system. The zeta potential value serves as an indicator of the extent of electrostatic repulsion between adjacent particles. In general, nanoparticles with a high absolute value of zeta potential possess a significant charge repulsion force among particles, making them less prone to coagulation and resulting in a relatively stable solution [11]. It is widely accepted that nanoparticle dispersions with zeta potential within ±20–30 mV are considered relatively stable [35]. The ZP of LPC-LP is −20 ± 2.18 mV, indicating that the liposome had excellent stability. The studies mentioned above indicate that ISL-LP exhibits desirable particle sizes, satisfactory stability, and high dispersion. Furthermore, it can be inferred that ISL-LP can prolong the circulation of ISL in the body and be more readily absorbed by the cells.

The in vitro release study is an essential process in the development of pharmaceutical formulations and the quality control of pharmaceutical products. It is an indicator of the stability and consistency of pharmaceutical preparations and can also be employed as an alternative for bioequivalence evaluation [36]. In order to simulate the in vivo conditions, the in vitro release profiles of free ISL solution and ISL-LP with equal doses were investigated in a phosphate buffer solution containing 1% SDS (pH = 7.4, 37 °C). The results are shown in Figure 2. Within the initial eight-hour period, nearly 90% of ISL was released from the free ISL solution, whereas the cumulative release from ISL-LP was only 43%. Afterward, ISL-LP released more slowly over the following 40 h, reaching a cumulative release of 60.31% at 48 h. The biphasic release behavior of ISL-LP observed in this study is mainly attributed to the initial burst effect, which is likely caused by the presence of free ISL on the surface of ISL-LP [37]. The sustained release process of ISL-LP is the diffusion of ISL through the polymer matrix. There is no doubt that the drug release is controlled by the matrix materials. The liposome membrane is a rate-limiting barrier, causing a delay in the diffusion of the drug, thereby creating a rate-controlled system. Therefore, it can be inferred that after intravenous administration of ISL-LP, ISL is gradually released into the blood, leading to an extended circulation time of the drug in the body. This, in turn, results in an increased concentration of the drug in brain microvessels and enhances its efficacy in targeting the brain.

Several mathematical models have been developed to fit the dissolution data and analyze the mechanism of drug release. The selection of a proper model is essential for the evaluation of drug release properties and the comparison of different release profiles [38]. In this paper, the drug release data from ISL-LP was processed by the DDsolver software 1.0. Four different release models were used to fit the release profiles of ISL-LP, including Zero-order, First-order, Weibull, and Higuchi kinetic models. The model equations are shown in Table 3, where “t” represents the corresponding time, “*F*” signifies the percentage of cumulative release, and R^2^ denotes the coefficient of determination, which is used to determine the most suitable model. The best-fitted model was found to be the Weibull distribution model with the maximum R^2^ value (R^2^ = 0.964). Therefore, it can be stated that the in vitro release of ISL-LP follows Weibull’s model to achieve a sustained release effect.

### 3.3. Pharmacokinetic Profiles of ISL and ISL-LP

**Figure 3:**
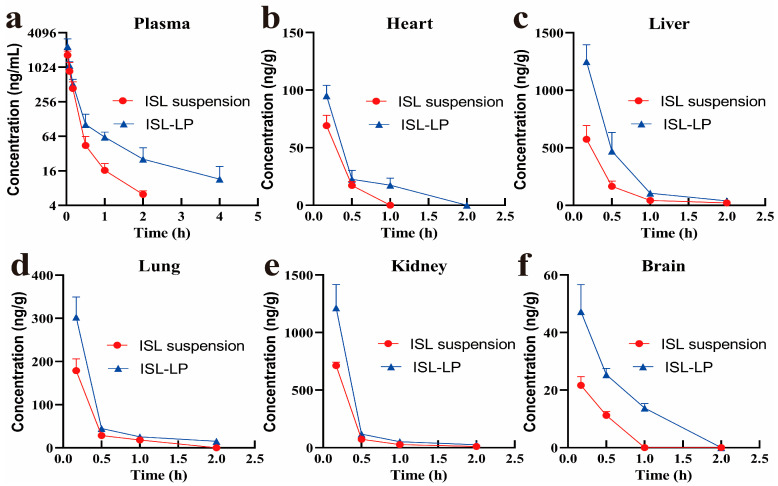
(**a**) The pharmacokinetic profile of ISL in SD rats after a single intravenous administration of ISL suspension and ISL-LP at a dose of 2 mg/kg (*n* = 6). The tissue distribution of ISL in the heart (**b**), liver (**c**), lung (**d**), kidney (**e**), and brain (**f**) after a single intravenous administration of ISL suspension and ISL-LP (2.8 mg/kg) to ICR mice (*n* = 4).

After intravenous administration of ISL-LP and ISL suspension at a single dose of 2 mg/kg in SD rats, the plasma concentration–time curves of ISL were plotted, as shown in Figure 3a (*n* = 6). In comparison to the ISL-LP group, the ISL suspension showed a lower average concentration of ISL in the plasma at each time point and was not detectable at 2 h after administration, whereas the plasma concentration of ISL in ISL-LP groups could be quantified for a longer time. Kinetic models were fitted to the plasma concentration of ISL over time by DAS 2.0 software. Subsequently, the pharmacokinetic parameters were calculated according to the statistical moment and compartment model, as shown in Table 4. The plasma concentration profiles of both formulations presented an initial rapid distribution phase followed by a slow elimination phase. The elimination half-life (*t*_1/2β_) of ISL-LP was found to be longer than that of ISL suspension, with a statistically significant difference (*p* < 0.05). In addition, the mean residence time (MRT) of ISL-LP was significantly prolonged by 2.6 times. This suggests that ISL-LP has a longer circulation time in the body compared to ISL suspension. The clearance rate (CL) of the ISL-LP was also significantly lower than that of the ISL suspension, with a statistical difference (*p* < 0.05). The area under the concentration–time curves (AUC) of ISL-LP and ISL suspension were 496.19 ± 126.29 μg·h·mL^−1^ and 306.78 ± 92.53 μg·h·mL^−1^, respectively, suggesting the relative bioavailability of ISL-LP was about 162%. The results demonstrated that ISL-LP significantly altered the pharmacokinetic profile of ISL in rats. It extended the drug’s circulation time, increased its exposure amount in vivo, and improved its bioavailability.

The differences in the pharmacokinetics of ISL between ISL-LP and ISL suspension are primarily attributed to the intrinsic properties of liposome nanoparticles in vivo and the surface modifications of liposomes. Liposomes are widely acknowledged for their good biocompatibility and low toxicity. It can alter the drug’s physical and chemical properties, such as its solubility, promote drug absorption, and control drug release [39,40]. However, liposomes containing phospholipids and cholesterol exhibit a high systemic clearance rate. They can be rapidly phagocytosed by the reticuloendothelial system (RES) in various organs such as the liver, spleen, bone marrow, and other tissues after intravenous administration. This significantly restricts their clinical application. The surface modification of liposomes with PEG can overcome this limitation [41]. PEG possesses amphiphilic properties and excellent biocompatibility, making it a popular choice for surface modification of nanoparticles to form a long-term circulation system in the body. PEGylation of liposomes leads to the formation of a hydration barrier on the surface of liposomes, which restricts spatial interaction with plasma proteins and prevents the recognition and phagocytosis of liposomes by the reticuloendothelial system (RES). This stealth effect significantly enhances the stability of liposomes in the bloodstream and extends the systemic circulation time, thereby prolonging the dosing interval and improving the drug bioavailability [42].

### 3.4. Tissue Distribution and Targeting Evaluation of ISL-LP

In order to explore the brain-targeting activity of ISL-LP in vivo, the concentrations of ISL in tissues were determined after intravenous administration of ISL-LP and ISL suspension to ICR mice. The tissue distribution of two formulations in mice is shown in Figure 3b–f (*n* = 4). The concentration of ISL in all tissues tested in this study increased to some extent compared with the ISL suspension, particularly in the brain. After 1 h of administration of the ISL suspension, complete elimination of ISL was observed in both the brain and heart. Additionally, the concentration of ISL in other tissues was found to be negligible. In contrast, the ISL-LP, as a long-circulating system, facilitated a prolonged presence of ISL in the tissues, which is consistent with its pharmacokinetic properties. Table 5 presents the AUC and targeting parameters for both formulations. Nanoparticulate systems possess the capability to alter the distribution of drugs in the body, thereby increasing their accumulation in specific target tissues. The parameters listed in Table 5 are critical indicators for evaluating the targeting activity of nanoparticles. TI is the ratio of the AUC of ISL-LP to that of the ISL suspension in tissues. It reflects the effect of ISL-LP on the uptake of ISL into tissues. It was observed that after the administration of ISL-LP, the TI values were ranked as follows: brain > liver > kidney = lung. ISL-LP could increase the AUC of ISL by approximately 3.45-fold in the brain tissue compared to the ISL suspension. TE represents the target organ selectivity of both nanoparticles and free drug solution, whereas RTE indicates the relative extent of change in this selectivity, with positive values indicating improved tissue targeting. Following intravenous administration of ISL-LP, the TE of tissues changed compared with the ISL suspension, with an increase in the brain and liver, particularly in the brain. The TE of the brain tissue in the ISL-LP group was 1.66 times higher than that of the ISL suspension group. In addition, the RET values of ISL-LP revealed that the brain tissue had the highest positive RTE value (65.64%), followed by the liver (16.22%), whereas the kidney, lung, and heart displayed negative RTE values. These findings mentioned above provide evidence that ISL-LP could enhance the relative uptake of ISL by the brain, indicating its potential as a delivery system targeting the brain.

The BBB plays a crucial role in maintaining internal homeostasis within the brain by protecting it from the adverse effects of harmful substances [43]. Nevertheless, this barrier property also restricts the delivery of pharmaceuticals to brain tissue. Although certain lipid-soluble drugs can penetrate the BBB, they are rapidly eliminated from the brain by efflux transporter systems, such as P-glycoprotein (P-gp). This significantly limits the effect of therapeutic drugs on the brain [25]. Therefore, there remains a significant challenge in delivering neurotherapeutic drugs through the BBB to treat neurological disorders efficiently. In order to overcome these limitations, we used PEGylated liposomes modified with angiopep-2 as carriers for the targeted delivery of ISL to the brain. The enhanced accumulation of ISL-LP in the brain can be attributed to several factors. Firstly, the liposome is a natural drug delivery system. The main components of liposomes are phospholipids and cholesterol, which are also the primary constituents of biomembranes with high biocompatibility and low toxicity [39,40]. Therefore, liposomes can transport encapsulated drugs more efficiently across the lipophilic endothelial barrier through endocytosis, achieved by fusing with cell membranes, as illustrated in Figure 1. This process allows the drugs to bypass the brain’s efflux pump system, thereby decreasing the amount of the drug eliminated from the brain [28]. In addition, drugs encapsulated in lipid bilayer-enclosed spherical vesicles are released slowly and continuously in vivo. This sustained release system reduces the degradation of the encapsulated drugs by plasma enzymes, thereby decreasing plasma clearance and enhancing their delivery to the brain [44]. Furthermore, the PEG coating on the surface of liposomes increases their “stealth” property [42]. PEGylation of the nanoparticle can improve the pharmacokinetic profile of the drug and prolong its circulation time in the body by inducing a steric hindrance effect to inhibit RES elimination [45]. The prolonged duration of nanomedicine in the brain capillaries leads to an increased concentration gradient, thereby enhancing the passive targeting efficacy of the nanoparticle. This process facilitates the drug’s permeation into the endothelial cell layer, thereby enhancing its ability to traverse the BBB [27,28]. In addition, the brain-targeting efficacy of nanoparticles can be enhanced by modifying the terminal of the PEG coating on their surface with bioactive ligands, including peptides and antibodies. These ligands can bind to receptors abundantly expressed on the BBB to successfully deliver nanoparticles to the brain through receptor-mediated transport. In this study, angiopep-2, a brain-targeting ligand, was conjugated to the terminal of PEG through an addition reaction. Angiopep-2 exhibits a high affinity for LRP-1, which is highly expressed in the brain [23,46,47]. Angiopep-2 conjugated on nanoparticle surfaces still maintains a high level of endocytosis mediated by LRP, thereby facilitating the transportation of drugs into the brain [46]. This transcellular transport process also bypasses the efflux protein system [48]. Furthermore, the long circulation system facilitates the ligand-receptor interaction by prolonging the duration of contact between angiopep-2 and LRP-1, thereby enhancing drug accumulation in the brain [48]. The LRP-1 receptor is also highly expressed on hepatocytes and is responsible for the transport of free fatty acids. This explains the increased RTE of the liver as observed in the distribution study [46]. Furthermore, the size of nanoparticles is also a very important factor in influencing the penetration of drugs into the brain. It has been reported that nanoparticles smaller than 100 nm in size are more likely to penetrate the endothelial cells of the BBB [30]. In this study, the size of ISL-LP is 89.36 ± 5.04 nm, further supporting its potential to target the brain. In conclusion, based on the factors mentioned above, ISL-LP is distributed sustainably into brain tissue, resulting in enhanced drug exposure in the brain and consequently enhancing its cerebral protective effects.

## 4. Conclusions

In the present study, a novel ISL-loaded liposome modified with a brain-targeting peptide was developed to overcome the defects of ISL, such as its inadequate water solubility, short half-life, and poor brain distribution. The optimized formulation of ISL-LP demonstrated advantageous characteristics, including a small particle size of 89.36 ± 5.04 nm, a polydispersity index of 0.17 ± 0.03, a zeta potential of −20.27 ± 2.18 mV, and a high encapsulation efficiency of 75.04 ± 3.28%. Further, in vitro release experiments indicated that ISL-LP was an effective sustained-release system. Pharmacokinetic studies showed that ISL-LP had the benefits of a significantly longer elimination half-life, lower clearance, and enhanced bioavailability compared to the ISL suspension. These findings distinctly illustrated that ISL-LP substantially improved the pharmacokinetic profile of ISL and prolonged its systemic circulation. The results of tissue distribution further elucidated the effectiveness of ISL-LP in targeting and delivering ISL to the brain, with the highest TI value in the brain. This targeted delivery was further corroborated by the highest positive RTE value (65.64%) in the brain. In summary, ISL-LP demonstrated significant potential as a promising brain-targeting system for the treatment and prevention of central nervous system (CNS) disorders.

Nevertheless, the clinical application of ISL-LP still faces many challenges. The precise cerebral distribution after penetrating the BBB remains unconfirmed, and the safety profile in non-target tissues requires further verification. Moreover, physiological differences between the model animals employed in studies and humans may lead to variations in drug behavior, affecting the potential for preclinical research findings to translate into clinical applications. Additionally, research on ISL-LP is still at the basic experimental stage, the systemic stability of liposomes requires further testing, and scaling up production remains challenging. These obstacles severely restrict the clinical advancement of ISL-LP.

To address these challenges, it is imperative to refine our understanding of drug distribution within the brain, conduct extensive toxicity studies, and evaluate long-term safety. Moreover, developing more accurate models and methods to simulate the behavior of nanomedicines within the human body remains a key direction for the development of this field. In terms of manufacturing and scale-up, further evaluation of the physical and chemical stability of ISL-LP under different storage conditions and durations and optimization of production processes are essential to ensure the uniformity and stability of nanocarrier systems. Additionally, it is also necessary to expand our research to compare the isoliquiritigenin-loaded plain liposomes with the liposomes modified with polypeptide to assess the specific enhancements offered by the peptide modification and then improve the preclinical evaluation system of nanomedicines. These measures lay a solid foundation for the clinical application of nanomedicines.

## Figures and Tables

**Table 1:** The factor-level design of an orthogonal experiment.

Levels	Factors		
	A	B	C
	PC (mg)	PC: Chol (mg/mg)	(PC + Chol): ISL (mg/mg)
1	3	3:1	10:1
2	6	6:1	20:1
3	9	9:1	30:1

**Table 2:** The results and analysis of the orthogonal experiment L9 (3^4^).

No.	Factors			EE%
	A	B	C	
	PC (mg)	PC: Chol (mg/mg)	(PC + Chol): ISL (mg/mg)	
1	3	3:1	10:1	29.23
2	3	6:1	20:1	58.29
3	3	9:1	30:1	46.95
4	6	3:1	20:1	62.09
5	6	6:1	30:1	73.86
6	6	9:1	10:1	50.84
7	9	3:1	30:1	73.01
8	9	6:1	10:1	62.49
9	9	9:1	20:1	77.74
K1	44.82	54.78	47.52	
K2	62.26	64.88	66.04	
K3	71.08	58.51	64.61	
R	26.26	10.1	18.52	

K1, K2, and K3 represent the mean values of the corresponding levels for each factor. R is the extreme difference of each factor.

**Table 3:** The models and regression equations for fitting drug release data from ISL-LP.

Model	Equation	R^2^
Zero-order	*F* = 1.759t	0.8008
First-order	ln(1 − *F*/100) = −0.04t	0.9231
Higuchi	*F* = 10.913t^0.5^	0.9216
Weibull	ln[ln[1/(1 − *F*/100)]] = 0.537lnt − 1.929	0.9640

**Table 4:** Pharmacokinetic parameters of ISL after single i.v. administration of ISL suspension and ISL-LP (2 mg/kg) to rats. (*n* = 6, mean ± SD).

Parameters	Unit	ISL Suspension	ISL-LP
*t*_1/2α_	h	0.07 ± 0.01	0.07 ± 0.02
*t*_1/2β_	h	0.70 ± 0.22	1.37 ± 0.52 *
AUC(0→t)	μg/L·h	306.78 ± 92.53	496.19 ± 126.29 *
AUC(0→∞)	μg/L·h	372.75 ± 105.57	607.59 ± 168.79 *
MRT(0→t)	h	0.17 ± 0.03	0.45 ± 0.13 **
CL	L/h/kg	5.78 ± 1.74	3.49 ± 0.88 *

* *p* < 0.05 and ** *p* < 0.01 vs. the ISL group; *t*_1/2α_ represents the distribution half-life; *t*_1/2β_ signifies the elimination half-life; AUC refers to the area under the plasma concentration-time curve; MRT denotes the mean residence time; CL represents the clearance rate.

**Table 5:** Targeting parameters of ISL in different tissues after intravenous administration of ISL-LP and ISL suspension in mice. (*n* = 4, mean ± SD).

Tissue	AUC (ng·h/g)		TE (%)		RTE (%)	TI
	ISL Suspension	ISL-LP	ISL Suspension	ISL-LP		
Heart	18.66 ± 2.11	37.58 ± 3.72	4.02	3.88	−3.30	2.01
Liver	207.70 ± 28.50	502.70 ± 65.75	44.70	51.95	16.22	2.42
Lung	55.24 ± 6.28	95.81 ± 9.67	11.89	9.90	−16.72	1.73
Kidney	174.7 ± 9.70	302.80 ± 40.40	37.60	31.29	−16.77	1.73
Brain	8.34 ± 0.61	28.77 ± 2.22	1.79	2.97	65.64	3.45

TE refers to the targeting efficiency; RTE represents the relative targeting efficiency; TI denotes the targeting index.

## Data Availability

Data are contained within the article.
